# Key considerations for applying intersectionality theory to partner and stakeholder engagement in public health

**DOI:** 10.17269/s41997-025-01023-7

**Published:** 2025-04-10

**Authors:** Samantha Ghanem, Nidhi Marulappa, Vivian Qiang

**Affiliations:** Public Health Agency of Canada, Ottawa, ON Canada

**Keywords:** SGBA Plus, Intersectionality, Stakeholder engagement, Partner engagement, Health equity, ACSG Plus, Intersectionnalité, Engagement des intervenants, Engagement des partenaires, Équité en santé

## Abstract

**Setting:**

Partner and stakeholder engagement (PSE) in public health that involves tokenistic and performative practices results in further marginalization of priority populations. Inclusive, intentional, and mutually respectful engagement requires an intersectional approach to account for the overlapping and compounding impacts of multiple systems of power on the lived experiences of priority populations. However, there is a lack of practical guidance on methods, strategies, or approaches for how public health initiatives can meaningfully engage with priority populations.

**Intervention:**

An evidence synthesis on intersectional approaches to PSE in public health was conducted to inform the development of an evidence-informed tool. Engagement approaches were evaluated based on (1) integration of intersectionality, as defined by the Intersectionality-Based Policy Analysis Framework and (2) level of stakeholder engagement achieved, from communication to co-production.

**Outcomes:**

The resulting tool offers “key considerations” for incorporating intersectionality principles in PSE in public health, encouraging critical reflection on the who, why, and how of PSE. Organized by the development, implementation, and monitoring and evaluation phases of public health initiatives, these considerations guide users through critical reflection by posing open-ended questions.

**Implications:**

The tool’s “key considerations” are relevant for all public health practitioners, with an emphasis on those in public health institutions. It guides users in navigating structural and interpersonal power imbalances with systemically marginalized priority populations. Adopting an intersectional lens enhances the ability to identify and address the complex array of determinants of health, tailored to population-specific needs and priorities.

## Résumé

**Contexte:**

La mobilisation des partenaires et des intervenants (MPI) dans le domaine de la santé publique, lorsqu’elle consiste en des pratiques purement symboliques et en une diversité de façade, entraîne une marginalisation accrue des populations prioritaires. Une mobilisation inclusive, motivée par une intention sincère et mutuellement respectueuse nécessite une approche intersectionnelle permettant de tenir compte des conséquences superposées et cumulées des multiples systèmes de pouvoir sur les expériences vécues par les populations prioritaires. Cependant, l’orientation pratique est lacunaire quant aux méthodes, aux stratégies ou aux approches qui permettraient aux initiatives de santé publique d’avoir un effet concret sur les populations prioritaires.

**Intervention:**

Une synthèse des données probantes sur les approches intersectionnelles à l’égard de la MPI dans le domaine de la santé publique a été réalisée afin d’éclairer la mise au point d’un outil fondé sur des données probantes. Des stratégies de mobilisation ont été évaluées en fonction de ce qui suit: 1) l’intégration de l’intersectionnalité, telle qu’elle est définie par le cadre d’analyse des politiques fondé sur l’intersectionnalité; 2) le niveau de mobilisation des intervenants, de la communication à la coproduction.

**Résultats:**

L’outil qui en résulte propose des «éléments clés à considérer» pour l’intégration des principes d’intersectionnalité dans la MPI dans le domaine de la santé publique et encourage une réflexion critique sur les partenaires et les intervenants qui sont mobilisés, la raison de leur mobilisation et la façon dont ils sont mobilisés. Organisés en fonction des phases d’élaboration, de mise en œuvre, de suivi et d’évaluation des initiatives de santé publique, ces éléments guident les utilisateurs dans leur réflexion critique au moyen de questions ouvertes.

**Conséquences:**

Les «éléments clés» de l’outil sont pertinents pour tous les praticiens de la santé publique, et plus particulièrement pour ceux qui travaillent dans des établissements de santé publique. L’outil guide les utilisateurs dans la gestion des déséquilibres de pouvoir structurels et interpersonnels avec les populations prioritaires systématiquement marginalisées. L’adoption d’une perspective d’intersectionnalité améliore la capacité de déterminer et d’aborder l’éventail complexe de déterminants de la santé, en tenant compte des besoins et des priorités de la population concernée.

## Setting

Meaningful partner and stakeholder engagement (PSE) with various audiences, including decision-makers, service providers, and priority populations, is crucial for equity-informed public health policy, programming, and research (Petkovic et al., 2020). Inclusive, intentional, and mutually respectful engagement requires an intersectional lens to understanding the lived and living experiences and unique needs of priority populations. Priority populations are communities who are placed at greater risk of adverse health outcomes due to overlapping and intersecting systems of oppression, including persons with disabilities, 2SLGBTQI + populations, racialized people, and First Nations, Inuit, and Métis (Government of Canada, 2023). In response to ongoing underrepresentation of priority populations in PSE activities, advocacy efforts for the inclusive participation in decision-making processes have long existed in public health. Notably, the emergence of the global disability movement in the late twentieth century put forth the central motto, “nothing about us, without us”, expressing that persons with disabilities know what is best for them and their communities (Sebring et al., 2022). This reinforces the need for meaningful PSE to inform equity-informed decision-making in public health, which operationalizes an intersectional lens, by looking both within and across priority populations (Government of Canada, 2023).

Originating from Black feminist studies, intersectionality theory acknowledges that systems and structures of power, such as racism, sexism, classism, and ableism, do not operate independently (Crenshaw, 1989). Instead, they intersect and overlap, to create distinct experiences of privilege and discrimination based on one’s multi-dimensional identities and social positionality, such as race, ethnicity, gender, socio-economic status, and disability (Crenshaw, 1989). Public health policy, programming, and research have historically reinforced systems of power and oppression against priority populations, and continue to do so (Datta et al., 2021). Canada’s colonial past, grounded in structural racism against Black, Indigenous, and other racialized populations, has perpetuated inequities in health, resulting in a justified lack of trust and willingness to participate in public health policy, programming, and research (Datta et al., 2021). An intersectional approach to PSE prompts public health practitioners to engage in critical self-reflection and practice cultural humility, while increasing accountability on the use of PSE feedback and expertise to guide public health policy, programming, and research that benefit all.

As per the Government of Canada’s Health Portfolio Sex- and Gender-Based Analysis Plus (SGBA Plus) Policy, the Public Health Agency of Canada (PHAC) is committed to advancing intersectionality and health equity through meaningful PSE in public health initiatives (Government of Canada, 2023). This entails considering the lived and living experiences and needs of people (Government of Canada, 2023). SGBA Plus prompts public health stakeholders to involve priority populations throughout the planning, implementation, monitoring, and evaluation stages of initiatives (Government of Canada, 2023). 

However, there is a lack of evidence-informed, practical guidance on methods, strategies, or approaches for how public health practitioners can engage with priority populations through an intersectional lens. For example, a review of Indigenous health literature found that although the proportion of articles reporting participatory, community-based methods increased over time, only 19% of examined studies demonstrated engagement with Indigenous Peoples, signalling tokenistic and performative practices (Jones et al., 2018). To address this gap, evidence on the effectiveness of PSE approaches through an intersectional lens was synthesized and analyzed, informing the identification of key considerations for conducting meaningful PSE with priority populations.

## Intervention

Evidence on PSE approaches with priority populations was synthesized and analyzed by all authors using the Intersectionality-Based Policy Analysis (IBPA) Framework, which is a set of eight guiding principles and a list of overarching questions to structure the analysis (Table 1) (Hankivsky et al., 2014). All authors conducted screening of evidence in two phases: phase one consisted of reviewing titles and abstracts and phase two entailed full-text review. For both phases, all authors discussed all discrepancies to clarify any differences in the application and interpretation of the eligibility criteria.

The IBPA Framework has been applied to a variety of public health policies and contexts to evaluate their impacts on priority populations (Hankivsky et al., 2014). Given the lack of comprehensive intersectional analyses conducted on PSE in public health, the IBPA Framework presents a promising approach for systematically identifying gaps in capacity for equity-informed practice within this domain. Following the analysis using the IBPA Framework, all authors assessed the articles for the level of PSE achieved as per the four levels of engagement (Table 2) (Petkovic et al., 2020).

**Table 1:** IBPA Framework guiding principles

Guiding principle	Overarching question
1. Intersecting categories	Did the authors demonstrate how social categories interact and intersect to create unique positionalities?
2. Multi-level analysis	Did the authors consider the multi-level determinants of health across various levels of society (global and national, provincial, territorial, and community)?
3. Power	Did the authors consider how systems and structures of power create and enable inequities?
4. Equity	Did the authors conceptualize, measure, or describe inequities between population groups at the intersection of multiple positions and systems of power?
5. Social justice	Did the authors integrate concepts of fairness, justice, and advocacy, as well as the need for policy or systems change?
6. Time and space	Did the authors integrate notions of temporal or spatial variation of sociopolitical contexts, experiences, risks, or outcomes?
7. Diverse knowledge	Did the authors acknowledge and consider the perspectives of diverse voices and communities in any component of the study design?
8. Reflexivity	Did the authors practice critical self-awareness and acknowledge how their experiences and privileges may influence their work?

Source: Hankivsky et al. (2014)

**Table 2:** Levels of engagement

Level of engagement	Description
Level 1 — communication	This involves stakeholders receiving information from the program leads but having no role in contributing to the initiative.
Level 2 — consultation	This allows for stakeholders to provide their views, thoughts, feedback, opinions, or experiences on the initiative, but program leads do not establish any commitment to act on them.
Level 3 — collaboration	This involves engaging stakeholders in influencing the development of the initiative (e.g., commenting, advising, ranking, voting, prioritizing, and reaching consensus) but without direct control over decisions.
Level 4 — co-production	This involves engaging stakeholders as equal members of the initiative development team and having them participate in all steps of the development, implementation, and reporting process.

Source: Petkovic et al. (2020)

All included studies were peer-reviewed, were based in Canada and aimed to apply an intersectionality-related framework or concept, were engaged with community-based partners and stakeholders for a specific public health program, intervention, or policy, and provided evidence for the effectiveness of the intersectional approach. These studies informed the identification of key considerations for conducting meaningful PSE with priority populations. Adopting PHAC’s consultation process, findings were validated through consultations with program areas across PHAC and several other government departments that regularly undertake PSE. Validation included written and verbal feedback elucidating how the results compare with current practices, as well as the utility of the key considerations.

## Outcomes

Twenty-one studies with diverse priority populations, initiatives, and PSE approaches were considered (Table 3). All studies examined outcomes in priority populations who experience inequities across multiple determinants of health, including Indigenous communities (First Nations, Inuit, and Métis), 2SLGBTQI + populations, racialized people, immigrants, people with disabilities, and people with lived and living experiences with financial and housing insecurity (Logie et al., 2019; Pakhale et al., 2016; Ratelle et al., 2018; Raynault et al., 2020; Sebring et al., 2022). Nine distinct PSE approaches were identified, including community-based participatory action research (CBPAR), peer researchers, community partnerships and consultations, advisory groups, focus groups, one-on-one interviews, workshops, surveys, and Indigenous methods (e.g., sharing circles, smudging) (Table 3). Below we discuss a few of the commonly adopted PSE approaches. All considered studies applied at least two engagement approaches and an intersectionality-related framework or concept to PSE, supporting the establishment of trusting relationships and empowering communities to design, implement, and sustain equity-informed public health initiatives. A meaningful outcome observed across studies is the multi-year implementation of culturally relevant initiatives tailored to community-specific needs, priorities, and practices.

**Table 3:** Mapping of stakeholder engagement approaches with corresponding levels of engagement

Study (reference)	CBPAR	Peer researchers	Community partnerships and consultations	Advisory groups	Focus groups	One-on-one interviews	Workshops	Surveys	Indigenous methods
(Born et al., 2012)	Level 4		Level 4		Level 4				Level 4
(Bottorff et al., 2018)	Level 3				Level 3	Level 3			
(Baskerville et al., 2018)			Level 2		Level 2				
(Charania & Tsuji, 2012)	Level 4			Level 4		Level 4			
(Charania & Tsuji, 2013)	Level 3			Level 3		Level 3			
(Gaspar et al., 2019)	Level 4	Level 4							Level 4
(Gerlach et al., 2017)			Level 2			Level 2			
(Gibson-Wood et al., 2012)			Level 3		Level 3	Level 3			
(Hamel-Smith Grassby et al., 2021)			Level 2		Level 2	Level 2			
(Kelley et al., 2018)	Level 4			Level 4	Level 4	Level 4	Level 4	Level 4	
(Logie et al., 2019)			Level 4		Level 4				
(Martin et al., 2018)			Level 2	Level 2	Level 2			Level 2	
(Pakhale et al., 2016)	Level 3	Level 3	Level 3						
(Pfaff et al., 2021)					Level 2	Level 2			
(Ratelle et al., 2018)		Level 4	Level 4			Level 4	Level 4	Level 4	
(Raynault et al., 2020)					Level 3	Level 3			
(Sebring et al., 2022)	Level 3				Level 3			Level 3	
(Shan et al., 2014)					Level 2	Level 2			
(Webkamigad et al., 2020)				Level 2	Level 2	Level 2			Level 2
(Young et al., 2017)			Level 3			Level 3			Level 3
(Young et al., 2013)	Level 3		Level 3	Level 3	Level 3				Level 3

### Community-based participatory action research

CBPAR actively involves communities in all stages of the initiative, from defining the scope to implementation and monitoring. This approach prioritizes diverse application and equitable engagement, focusing on community needs and strengths (Born et al., 2012; Bottorff et al., 2018; Charania & Tsuji, 2012; Charania & Tsuji, 2013; Gaspar et al., 2019; Kelley et al., 2018; Pakhale et al., 2016; Sebring et al., 2022; Young et al., 2013). Key practices within CBPAR include establishing community advisory groups, hiring peer researchers with lived and living experiences, and forming Research Agreements to clarify roles and expectations. Advisory groups serve as an authoritative body with expertise to guide research decisions and ensure alignment with community values (Kelley et al., 2018). Peer researchers—individuals with lived and living experience—help reduce power imbalances by participating in various research phases (Pakhale et al., 2016). They are recruited and trained by the research team, who prioritizes ethical considerations throughout their involvement, including fair compensation and confidentiality, while creating a supportive environment that addresses power dynamics (Pakhale et al., 2016).

This approach effectively engages priority populations across multiple determinants of health, which incorporates the intersectionality principle of “intersecting categories.” “Diverse knowledge” is also inherent to the CBPAR framework, as the approach aims to centre the perspectives of diverse voices and communities, while actively engaging them throughout the process. Additionally, CBPAR aims to address the principles of “power” and “reflexivity,” as the framework recognizes systemic power structures within the context of research and seeks to minimize power imbalances between researchers and participants. CBPAR is particularly effective with Indigenous populations and marginalized groups as it prevents, to a certain extent, the reproduction of inequitable power relations through establishing collaborative partnerships and learning about and translating their values into action (Born et al., 2012; Gaspar et al., 2019; Young et al., 2013). Overall, CBPAR is effective for achieving high levels of collaboration and co-design, resulting in tailored public health initiatives and sustained partnerships.

### Community partnerships and consultations

The community partnerships and consultations approach involves collaborating with community-based organizations, local experts, and general community members. Examples of community partners include community grassroots organizations (Gibson-Wood et al., 2012; Pakhale et al., 2016), community leaders (Logie et al., 2019), and Indigenous community centres (Webkamigad et al., 2020; Young et al., 2017). Community partnerships can be established both within and independent of the CBPAR framework and are built over time to establish trust. This trust often develops through ongoing collaboration, transparency, and mutual understanding, especially when prior relationships or shared experiences are not already in place.

Studies employed community partnerships for various purposes, including informing the initiative’s priorities, design, and implementation, as well as participant recruitment. Ratelle et al. (2018) spent two years building relationships with First Nations communities before data collection, ensuring research aligned with community needs. Logie et al. (2019) involved transgender community leaders in intervention conceptualization and data analysis. Pakhale et al. (2016) collaborated with a grassroots organization to recruit individuals with lived experiences as peer researchers.

Most literature does not detail how partnerships are established and leveraged. Partnerships involve priority populations at various intersections of social identities, addressing the intersectionality theory principle of “intersecting categories.” It also inherently involves people with lived and living experiences, integrating “diverse knowledge,” and addressing “power” imbalances by co-designing research with community input. Community partnerships demonstrate all engagement levels from consultation to co-design, facilitating meaningful, ongoing relationships and culturally safe initiatives.

### Focus groups and interviews

Focus groups and interviews are the most commonly used methods for collecting qualitative data and exchanging information with participants. While interviews are most often conducted with one participant, focus groups allow for interaction and discussion between participants to explore and clarify beliefs, opinions, and views. Crucial to effective engagement is striving to conduct these sessions in safe, comfortable environments, especially for priority populations. For example, Baskerville et al. (2018) facilitated focus groups for 2SLGBTQI + youth in safe community spaces, led by facilitators from the same community, fostering a sense of belonging and trust. Similarly, culturally relevant practices, such as incorporating Indigenous traditions in sessions with First Nations communities and providing light snacks and refreshments, enhanced engagement, relationship-building, and trust (Webkamigad et al., 2020).

Studies effectively integrated the intersectionality principle of “intersecting categories” and incorporated the principle of “diverse knowledge,” as focus groups and interviews were conducted directly with people with lived and living experiences. However, a majority of studies that used these engagement approaches did not position participants as collaborators; rather, participants often served only as producers or sources of knowledge. The studies varied in engagement levels, from solely consultation to co-design. While some studies gathered feedback without actionable outcomes, others involving co-design led to significant impacts and culturally relevant program development.

### Workshops

Workshops engage stakeholders through group sessions aimed at structured discussion on specific issues to bridge differences in knowledge (Kelley et al., 2018; Ratelle et al., 2018). For example, one study conducted workshops with Elders and local leaders in First Nations communities in the Canadian subarctic to “clarify conceptual differences in language and the meaning of words between research and community members” (Ratelle et al., 2018). These workshops integrated local knowledge, aligning with the principle of “diverse knowledge,” and helped develop a culturally safe biomonitoring project for the communities (Ratelle et al., 2018).

### Surveys

Surveys can be used to gather stakeholder feedback alongside other methods and should be developed in collaboration with representatives from the priority population to ensure cultural relevance. In PSE, surveys can be used for various purposes, including collecting demographic information (Kelley et al., 2018; Martin et al., 2018; Sebring et al., 2022); understanding health-related knowledge, attitudes, and behaviours (Martin et al., 2018); and gauging community interest (Ratelle et al., 2018). However, surveys may limit opportunities for reciprocal knowledge exchange, potentially hindering the integration of intersectional principles of “diverse knowledge,” “power,” and “reflexivity.” This limitation could reinforce traditional power imbalances between public health practitioners and communities.

### Implications for intersectional analysis

Based on the analysis guided by the IBPA Framework, studies consistently applied several principles of intersectionality, including “intersecting categories,” “diverse knowledge,” “equity,” and “social justice.” However, existing PSE work lacks focus on “reflexivity.” Few researchers included a statement on their positionalities relative to research participants and the impacts of their presence and activities on diverse communities. This principle seeks to dismantle inequitable power structures and is central to intersectionality in public health practice. Without reflexivity, researchers risk reinforcing biases or overlooking the needs of priority populations, potentially leading to interventions that lack cultural relevance. In practice, “reflexivity” invites public health practitioners to reflect on how their knowledge, experiences, and values influence their work (Hankivsky et al., 2014). 

The analysis also revealed the heterogeneous nature of definitions, concepts, and frameworks related to intersectional analysis. Only one study explicitly defined intersectionality, others used adjacent principles and frameworks. For example, principles related to intersectionality included participatory design (Bottorff et al., 2018), structural racism (Hamel-Smith Grassby et al., 2021), and disability justice (Sebring et al., 2022). Among studies involving Indigenous Peoples, principles related to intersectionality included cultural competency, self-determination, and Two-Eyed Seeing, which is a framework that honours and respects both Indigenous and Western ways of knowing (Born et al., 2012; Gaspar et al., 2019; Webkamigad et al., 2020; Young et al., 2013, 2017). 

The variability in interpretation and application of intersectionality theory may stem from its divergent adoptions across various disciplines and sociopolitical issues (Hankivsky et al., 2014). Intersectionality aims to capture complex, intersecting social categories and their interactions within broader historical and societal systems. It does not offer a “one size fits all” methodology for analyzing health inequities but is meant to be conceptualized and implemented dynamically according to evolving sociopolitical contexts and community-specific needs.

The meaning and application of intersectionality remain ambiguous (Hankivsky et al., 2014), presenting challenges in effectively translating theory into practice. While some articles describe quantitative outcome measures, such as demographic information indicating participant diversity, others provide general details about public health initiatives. Future directions may include developing standardized methods to measure the impact of stakeholder engagement through an intersectionality lens. Employing concrete indicators of meaningful PSE would allow public health practitioners to be held accountable to standards of intersectionality and health equity.

### In practice: Key considerations for applying intersectionality theory to partner and stakeholder engagement in public health

Findings are presented in a series of practical questions, guiding users towards key considerations in conducting equitable and meaningful PSE with diverse priority populations (Table 4). Key considerations vary according to the specific needs and priorities of different contexts; what is deemed culturally relevant for one community may not apply to another (Kahan, 2012). As such, the list of key considerations should be used as a prototype that is adapted alongside community stakeholders, according to the specific needs and considerations of each engagement opportunity. This tool can be applied both prospectively, to guide the design, implementation, and monitoring and evaluation of initiatives, and retrospectively, to learn from previous initiatives, enabling continuous learning and improvement.

**Table 4:** Key considerations for applying intersectionality theory to partner and stakeholder engagement in public health

Initiative phase	Questions to consider
Development	Identify the issue: • Who should you consult or engage with to ensure that the initiative reflects diverse perspectives? • Does the identified issue affect some groups more than others within the community? How have you engaged all priority populations within the community in defining the issue? • What are the expressed needs and priorities of the community? How does the initiative address these needs? • Will some groups within the community benefit from the initiative more than others?
	Identify and engage with the community: • Who is the priority community of your initiative? • Who represents or speaks on behalf of the community? • Are there institutional, organizational, or other social dynamics that privilege some voices over others? • Is there a history of exploitation or harm perpetrated by external stakeholders within the community? How might this history affect your positionality? • Did you approach the community sufficiently in advance to develop a relationship before the start of your initiative? • Have you gained approval for your initiative from diverse partners and stakeholders within the community? • Have you ensured that the relationship is mutually beneficial between all partners and stakeholders involved? • What are the community’s strengths? How does the community intend to contribute their knowledge and expertise to the initiative? • How do you intend to share decision-making power with the community? • Are there opportunities to involve individuals with lived and living experiences from the priority community as co-leads of the initiative and compensate them financially for their time?
	Develop the initiative: • What are the levels of engagement you hope to achieve across the various stages of the program? • Have you involved community partners and stakeholders in designing each element of your initiative protocol? o Have you learned about and integrated the community’s needs and preferences throughout all aspects of your initiative? Did you verify that expectations were met with the community? o How will you ensure open and regular communication with community partners and stakeholders throughout the development, implementation, and monitoring and evaluation process? • Are there any barriers that may hinder engagement from diverse groups within the community and how can these be addressed? • How can the initiative design address existing systems of power?
Implementation	Implementation of engagement approach: • How will you maintain respect for an individual’s decision to engage or not engage in your initiative? • Have all engagement tools been co-developed and approved by community partners and stakeholders? • Will engagement sessions take place in accessible locations where all individuals feel comfortable and safe? • Have you aimed to mitigate any potential power dynamics between engagement facilitators and community partners? • How will you ensure that the engagement approach meets the cultural needs or preferences of the community? o Are there opportunities to humbly and respectfully learn about and incorporate the community’s traditional ceremonies or practices? • How will you ensure that the community participants are unharmed and gain as much benefit as possible through their engagement? • Will you use accessible approaches and processes to obtain diverse perspectives from the community? • Will you provide resources and support to community participants during and after engagement sessions?
Monitoring and evaluation	Information ownership, access, and dissemination: • Who owns and has access to the information collected? Did the community participate in making this decision? • Does the community have control over information management processes? • How will you share the information with the community? o Did you present the findings in ways that community members understand and find meaningful? o How is knowledge returned to the community? • How will you ensure that your research accurately represents the community’s lived and living experiences and knowledge? How will the community substantiate or verify the validity of your findings? • How will you protect the privacy and safety of community members? What consequences might partners and stakeholders experience if their identities are revealed?
	Evaluation: • At what level did you engage community partners and stakeholders during the initiative’s development, implementation, and evaluation? • How will you seek community feedback on their experiences with the initiative? How do you intend to respond to and act upon this feedback? • How will the community be fairly compensated for their work and contributions? o Who will receive recognition for the work conducted in the initiative? Did the priority community participate in making this decision? • Who will evaluate the initiative’s performance? • What are the initiative’s measures for success? How will the community contribute to defining these measures? • How will you maintain relationships with the community moving forward?

### Limitations

Three limitations were identified in relation to the evidence synthesis and accompanying key considerations. The search strategy for the evidence synthesis was restricted to studies that mentioned geographic location in Canada in the title, abstract, or keywords. This limits the generalizability of findings, as it excludes international perspectives and insights that could broaden the understanding of effective practices. Furthermore, the evidence synthesis did not include non-peer-reviewed literature, such as books and grey literature, which excludes perspectives from groups who are systematically equity-denied within academic spaces. It also precludes the full consideration of non-Western research methodologies and traditional knowledges that emphasize storytelling and lived experiences. Due to a lack of knowledge in the systematic evaluation of stakeholder engagement through intersectionality theory, our analysis does not follow peer-reviewed measures for quantitatively evaluating the construct of interest. However, we utilized the well-established and widely used Intersectionality-Based Policy Analysis Framework (Hankivsky et al., 2014), to guide our understanding and implementation of intersectionality. Future work may consider exploring additional approaches to assessing and standardizing the level of “success” achieved by stakeholder engagement methods.

## Implications

Applying intersectionality theory to PSE advances equity in public health by improving the capacity to address the complex array of determinants of health, tailored to population-specific needs and priorities. Public health practitioners must acknowledge that PSE efforts can burden priority populations and lead to engagement fatigue if communities are not meaningfully involved or their needs are unmet, potentially perpetuating power imbalances and exploitative practices (Brunger & Wall, 2016). It is important to elucidate the reasons for engaging with priority populations and ensure that tailored initiatives meet community needs and priorities, aligning with proportionate universalism. 

Establishing trustworthy relationships with priority populations takes time and is critical for equitable public health practice across all public health institutions. Although direct engagement with priority populations may not always be possible, these principles apply to partnerships with all stakeholder groups. For example, PHAC’s Mental Health of Black Canadians (MHBC) Fund established a MHBC Working Group of mental health practitioners, academics, researchers, and individuals with lived and living experience from Black communities across Canada (Public Health Agency of Canada, 2023). The Working Group played a leadership and advisory role, including providing strategic advice on project funding and support to MHBC funded projects, provided guidance on capacity building and knowledge mobilization, and strengthened evidence on the key determinants of health impacting Black communities (Public Health Agency of Canada, 2023). 

Incorporating intersectionality in PSE allows public health to move away from a paternalistic model to one that recognizes and respects that communities have a right to identify their own health priorities, and in collaboration with public health institutions, act upon them. The tool’s “key considerations” are relevant for all public health practitioners, with an emphasis on those in public health institutions. It guides users in navigating structural and interpersonal power imbalances with systemically marginalized priority populations, supporting equitable health outcomes.

## Implications for policy and practice

What are the innovations in this policy or program?

- The Tool “Key considerations for applying intersectionality theory to partner and stakeholder engagement in public health” operationalizes intersectionality in public health PSE, providing a structured approach for critical reflection on who is engaged, why, and how, thereby supporting public health practitioners to identify and address power imbalances with priority populations.
- By organizing “key considerations” across the development, implementation, and monitoring and evaluation phases, the tool offers a prototype for integrating equity throughout a public health initiative’s lifecycle.

What are the burning research questions for this innovation?

- What approaches can be developed to evaluate the success of incorporating intersectionality principles in public health PSE?

## Data Availability

Not applicable.
